# Impact of Platelet-Rich Plasma on Bone Height Changes around Platform Switched Implants Supporting Mandibular Overdentures in Controlled Diabetic Patients

**DOI:** 10.3889/oamjms.2015.126

**Published:** 2015-12-10

**Authors:** Eman Mostafa Ahmed Ibraheem, Amany Mohy Eldeen

**Affiliations:** 1 *Prosthodontics Department, National Research Center, Giza, Egypt*; 2 *Oral Surgery and Oral Medicine Department, National Research Centre, Cairo, Egypt*

**Keywords:** Dental implant, platform switching, platelet-rich-plasma, CBCT, crestal bone remodeling, osseointegration

## Abstract

**BACKGROUND:**

The platform switching concept was recently introduced to implant dentistry involving the reduction of restoration abutment diameter with respect to the diameter of dental implant. Long-term follow-up around these implants showed higher levels of bone preservation and proper stress distribution and improved esthetics.

**AIM:**

The aim of the present study was to evaluate the changes in bone height by means of radiographic examination around platform switched implant supporting mandibular overdentures in controlled diabetic patients.

**SUBJECTS AND METHODS:**

Fourteen male complete edentulous patients were selected and enrolled in a follow-up study plan. Split mouth technique was applied; one side implant chosen randomly with Platelet-rich-plasma (PRP) and the other without PRP, bone height changes was assessed by Cone Beam Computed Tomography (CBCT) radiographic examination after 3 months, 6 months, 9 months and 1 year later.

**RESULTS:**

There was increase in bone height loss in both sides but with no statistical significance difference between the two sides after 3 months, 6 months, 9 months and 1 year respectively.

**CONCLUSION:**

The result of this article satisfied the patients both esthetically and functionally with recorded increase in bone height loss.

## Introduction

Platelet-rich-plasma (PRP) is autologous conditioned plasma contain high concentration of platelets which include numerous growth factors that facilitate tissue repair and healing [1]. Growth factors are Transforming Growth Factor-β (TGF-β), Platelet Derived Growth Factor (PDGF), Insulin-like Growth Factor (IGF-1) Vascular Endothelial Growth Factor (VEGF), Epidermal Growth Factor (EGF) and Fibroblast Growth Factor (FGF). These growth factors range from blood vessels development and repair to cellular recruitment and its activation [2]. PRP have been used in both animal and human studies to enhance and promote soft tissue repair and bone regeneration. PRP impact cellular attachment, differentiation, proliferation and bone matrix deposition [3]. Anitua, 2008 [4] reported that osseointegration of implants enhanced by wrapping PRP on implant surface before its insertion into the alveolus and could be considered as a safe and predictable procedure. Correspondingly, Nikolidakis, 2008 [5] and co-workers remarked significant effect on bone apposition when PRP coated on roughened titanium implants in early phase of healing. The purpose of modern implant therapy entails more than successful osseointegration of implant but also it include an esthetical and functional restoration [6]. Osseointegration is the ultimate goal in implant dentistry for tissue regeneration and proper healing [7]. The presence of a micro gap at the implant-abutment interface resulting in bacterial colonization in the implant sulcus, causing bacterial leakage within the implant system and inflammatory process at crestal bone, subsequently, loss of bone support [8]. The prosthetic concept of platform switching using an abutment with smaller diameter than the diameter of implant shoulder allow the microgap to be located more distant to the first-bone implant contact [9]. The inward movement of IAJ (implant abutment junction) when using platform switching shifts the inflammation towards the central axis of implant and away from adjacent crestal bone [10]. The resorption resulting from biological processes after prosthetic restoration changed when using the platform switched model [11]. Platform switching improves long-term bone maintenance around implants [12].

The present study was conducted to investigate the effect of one of the most known autologous growth factor (PRP) around platform switched implants on crestal on bone height (level) with a one-year follow-up in completely edentulous controlled diabetic patients.

## Subjects and Methods

The study protocol was approved by Ethical Committee of the National Research Center, Cairo, Egypt.

Fourteen male completely edentulous patients, with age ranging between (45-60) years were selected from the out-patient clinic, Fixed and Removable Prosthodontic Department, National Research Centre, Cairo, Egypt. All patients were evaluated before inclusion in this study through history, clinical& radiographic evaluation. All the patients had been completely edentulous for at least 5 years, with an apparent detection of narrow inter-arch space. All patients were diagnosed as type-II diabetics as it is more common than type-l. Only controlled diabetic patients were enrolled as they were considered like healthy subjects as they have better healing of the tissues and less liability for infection than uncontrolled patients, thereby, helps osseointegration process. To be included in this study, patients were selected to have good physical and mental health, sufficient bone of good quality and quantity to support implants evaluated in digital panoramic radiographs to allow the use of 13 mm implants in the symphyseal area. Patients were excluded if they had diseases that might negatively influence implant installation &/or Osseo integration. All patients were thoroughly informed about the study and each signed a written informed consent form. Only motivated & cooperative patients who accepted the periodic recall visits were enrolled

### Prosthetic Procedures

Complete maxillary and mandibular dentures were fabricated for all patients prior to implant installation in the mandibular symphyseal area to ensure prosthetic driven implant placement in harmony with osseous anatomy, denture esthetics and abutment connection. Lingualized balanced occlusal scheme was verified clinically to ensure equal distribution of posterior occlusal contacts and no anterior contacts. Following denture placement and patient adaptation, the mandibular denture was duplicated in clear acrylic resin to be utilized as a surgical stent.

### Study Implants

Two titanium endosteal root-form Screwplant implants [Spectra-System, Implant Direct LLC, CA]. Implants of 13 mm length and 3.7 mm diameter were chosen to be installed in mandibular canine area. The implants have an internal connection with 2 mm long hex and external bevel with 3.7 mm diameter platform. The body of the implant is tapered evenly down to facilitate insertion with least heat generation.

### Platelet Rich Plasma (PRP) preparation

PRP was prepared one hour before the surgical phase. Ten ml of autologous venous blood was drawn of the anticubital vein. Blood was collected in a sterile tube containing 10% Tri-sodium citrate solution anticoagulant. The blood sample was introduced into a differential blood counter (Medonic CA, 6201530, Sweden) to count the platelets before PRP preparation. It was 237.0000/mm^3^.

**Figure 1:**
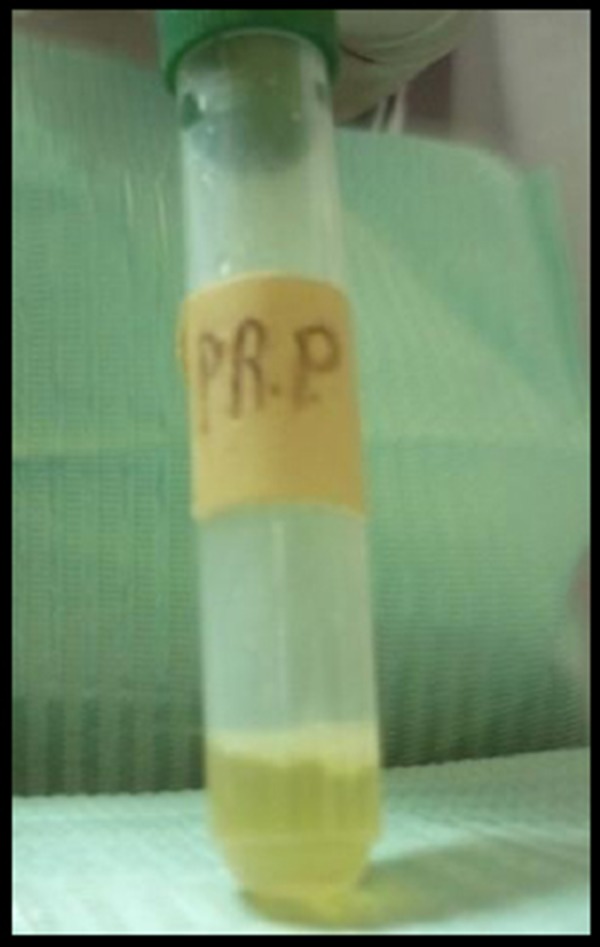
*Prepared PRP*.

The blood sample was centrifuged at 1500 rpm for 10 minutes in a centrifugal machine. After the first centrifuge, the red blood cells were separated from the top plasma which contained platelets and white blood cells. Plasma was drawn off the top into another sterile tube and centrifuged for additional 10 minutes at 3000 rpm. This centrifuge separates Platelet Poor Plasma (PPP) in the upper part and PRP in the lower part (visual differentiation between the two layers was difficult). PPP was discarded and PRP was introduced into the differential blood counter to count the platelets. The platelet count was increased about three times the pre-operative count to be 740.000/cm (Fig. 1).

Coagulation of PRP was achieved by a mixing it with 5cc sterile saline solution containing 10% calcium chloride and 5000 units sterile bovine thrombin (American Reagent Laboratories, NY) in a sterile dipping dish. Within few seconds, the sticky gel consistency was obtained.

### Surgical Procedures

Split mouth technique was followed in the present study; I.e. one implant bed was randomly chosen to receive PRP concomitant with the implant installation. However, the other bed implant was installed without PRP. Patients received professional oral hygiene measures prior to the operation and were instructed to rinse with a chlorhexidine mouthwash 0.2% for 1 minute, twice a day, starting 2 days prior to the intervention and thereafter for 2 weeks. All patients received prophylactic antibiotic therapy Amoxicillin Clavulinate 2 gm. 1 hour prior to the intervention. Aided by the surgical guide, implants were installed in the canine regions at equal distance from the mid line, parallel to each other and perpendicular to the occlusal plane. The prepared PRP was utilized to coat the implant to be installed in one quadrant. On the other hand, implant was installed in the other quadrant without PRP. The insertion torque values were set at 35 N-cm and the healing abutments were then threaded onto the implants to be utilized as overdenture abutments.

All implants were placed according to the one stage surgical protocol. The patient was recalled after seven days to remove the sutures Patients were instructed to avoid brushing and trauma at the surgical sites. Cold and soft diet was recommended for 7 days after surgery. During the initial healing period (two weeks after surgery) no prosthesis was used over the implants so that early healing can occur without functional loading. After the two weeks period, the tissue surface of the existing denture was relieved in the area overlying the installed implants to accommodate the newly inserted healing collars. Resilient relining material, (Permsoft Myerson Chicago IL USA) was placed into the relieved areas to assure intimate tissue contact. The dentures were adjusted, relined with a soft lining material (Riteline Rite, Dent. Mfg. Corp, USA) and the complete over denture was then checked intra orally for complete seating then immediately delivered to the patients. Osseointegration of the implants was verified by digital panoramic radiographs. Patients were recalled after 3 months for oral hygiene maintenance, prosthetic check-ups, removal of soft relining material and loading of the implants with ball attachement. Self -cured acrylic resin (Lucitone 199; Dentsply) was injected in the relief areas made opposite to the abutments positions. The complete overdenture was inserted in the patient’s mouth and close-mouth technique was carried to ensure intimate adaptation. After hardening of the acrylic resin, the denture was finished and polished. The dentures were inserted, and pressure indicating paste(MizzyInc, Cherry hill, NJ) was utilized to identify pressure areas and to ensure point contact with dome-shaped healing collars.

For the entire duration of the study (1 year after loading once every 3 month), the peri-implant marginal bone level changes were assessed with CBCT radiographs.

### Radiographic evaluation

**Figure 2:**
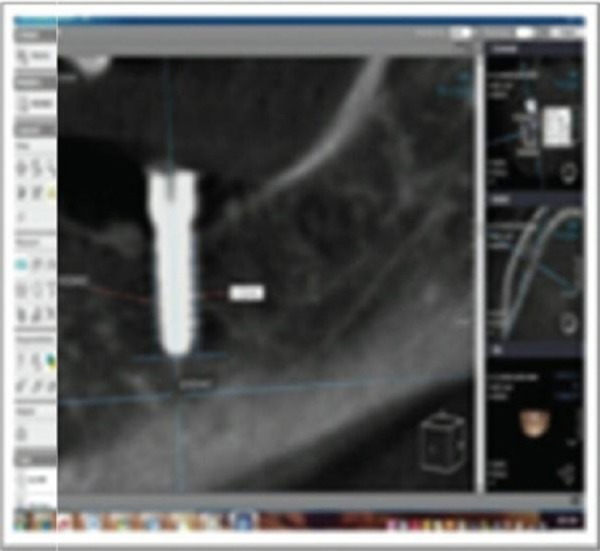
*Measuring bone height mesial and distal to the implant*.

Using Cone Beam Computed Tomography (CBCT) Images were acquired using the Scanora 3D Imaging system (Scanora 3D, Sorredex-Finland) (voxel size 133um-350 um) which allows the recording of linear bone height of images. The personal computer utilized was an Intel Core Duo- 2.13 Mhz- 3.25 Gbites-21 inches flt screen 9 Hewlett-Packard Pavilion Elite m9200t series (Hewlett-Packard Pavilion Elite m9200t series USA) Linear measurements for evaluation of crestal bone height, mesial and distal crestal bone levels were calculated from the reconstructed corrected sagital views by drawing a line parallel to the implant serration extending from the crestal bone to the apical end of the implant. Similarly, buccal and lingual bone levels were calculated by using cross-sectional views. Average readings of the four sides at each interval were calculated and tabulated for statistical analysis (Fig. 2).

## Results

### Comparing Bone height between the two sides

Comparison between the bone heights for both sides is shown in Table 1. We can see that there are no significant differences.

**Table 1:** Comparison between the bone heights for both sides

Period	Implant		Implant + PRP		*P*-value

	Mean	SD	Mean	SD
3 months	1.29	0.2	1.36	0.2	0.089

6 months	1.42	0.2	1.45	0.2	0.496

9 months	1.53	0.2	1.51	0.2	0.591

12 months	1.61	0.2	1.57	0.2	0.423

*: Significant at P ≤ 0.05

There was no statistically significant difference between mean bone height of the two sides after three months, and after six, nine and twelve months.

### Effect of time on each side

The effect of time and changes in the bone height for the implant side is shown in Table 2. There was a statistically significant increase in bone height loss through all observation periods.

**Table 2:** Changes in the bone height by time for the implant side

Period	Mean difference	SD	*P*-value
3 months – 6 months (After loading)	-0.13	0.1	0.004*

6 months – 9 months (After loading)	-0.11	0.06	<0.001*

9 months – 12 months (After loading)	-0.08	0.06	0.003*

* Significant at P ≤ 0.05

The changes in the bone height by time for the implant with PRP side are shown in Table 3. There was a statistically significant increase in bone height loss through all observation periods.

**Table 3:** Changes in the bone height by time for the implant with PRP side

	Period Mean difference	SD	*P*-value
3 months – 6 months (After loading)	-0.09	0.06	0.001*

6 months – 9 months (After loading)	-0.06	0.07	0.024*

9 months – 12 months (After loading)	-0.06	0.05	0.005*

* Significant at P ≤ 0.05

## Discussion

According to the results in the present study of one-year follow-up, there was a statistical significant increase in bone height loss in both treatment sides. The implant side results were similar to Astrand et al. 2004 [13] study that reported change in crestal bone-level. Cumbo et al., 2013 [10] concluded in a study that platform switching improves bone crest preservation thereby, achieved less rate of bone loss and better esthetics compared to conventional implant system. Reduction in crestal bone loss in platform switching achieved by shifting the inflammatory cell infiltrate inward and away from adjacent crestal bone and thus maintain the biological width and increase the distance of IAJ from crestal bone level and through the reduction of microgap on crestal bone [14]. Bone regeneration requires an orchestrated sequence of biological events of multifactorial regulations. Therefore, PRP an elected delivery system for growth and differentiation factors used in this study as it is of an autologous origin thus displaying no risk for transmission of disease and it plays an important role in bone formation [15]. Recent studies declared the promotion of bone regeneration and soft tissue healing when PRP applied on bone graft material [16]. There was statistical significant increase in bone height loss at the implant side group after 6 months, 9 months and 1 year follow-up respectively. Corresponding to the present study, Hϋrzeler et al., 2007 [17] observed bone remodeling 1 year follow-up after implant placement. The implant side with PRP showed a statistical significant increase in bone height through all treatment periods at 6 months, 9 months and 1 year respectively. The data of this study showed that PRP does not accelerate bone formation. The studies of Zechener et al., [18] and Marx et al., [19] support our results. PRP contribute optimizing and accelerating the osseointegration process through its osteoconductive properties attributed to fibrin and its osteoinductive activity [20]. Topical application of PRP during dental implant placement enhance early bone implant contact (BIC) [18], with minimal bone loss around the implants, same observation made by Glauser et al [21], Calandriello et al., [22] Abboud et al., [23] and Donati et al., [24] in their studies. Analyzing the results, there was no statistical significant difference between the two sides (implant side) and (implant with PRP side). This could be attributed to the primary stability gained during implant insertion which minimized the effect of PRP in bone formation around implants [18].

In conclusion, the randomized clinical trial reveals gain in bone height in using platform switching in dental implant. On the other hand, the effect of PRP was not beneficial to accelerate the osseointegration with the platform switching in dental implant.
